# Prolonged grief disorder following suicide loss – a narrative review

**DOI:** 10.1192/j.eurpsy.2026.12004

**Published:** 2026-07-31

**Authors:** A.-L. Comșa, I. C. Mândraș

**Affiliations:** 1 Psychology and Educational Sciences, Babes-Bolyai University; 2 Psychiatry, County Clinical Energency Hospital, Cluj-Napoca , Romania

## Abstract

**Introduction:**

Prolonged grief disorder (PGD) following suicide loss presents a distinct clinical challenge marked by persistent yearning, emotional pain, identity disruption, diminished sense of meaning and functional impairment. While these symptoms can arise in various bereavement contexts, suicide loss appears to be a particularly potent precipitant given its often traumatic nature and the complex interplay of emotional, social, and cognitive factors it triggers.

**Objectives:**

This synthesis examines epidemiological patterns, diagnostic criteria evolution, and differentiations from related disorders such as depression and PTSD, highlighting the elevated prevalence and unique symptom profiles among suicide-bereaved individuals.

**Methods:**

A database search was conducted in PubMed, ScienceDirect, and PsycINFO using a previously established search string. This process yielded 12 published articles meeting the inclusion criteria, with publication dates ranging from 2016 to 2025.

**Results:**

Epidemiological evidence consistently indicates elevated prevalence rates of PGD among suicide-loss survivors compared to other bereavement types, with particular vulnerability observed in close kinship roles. Psychological impacts include complex cognitive emotional processes involving guilt, self-blame, intrusive imagery, and stigma internalization, which interact with social and familial dynamics to influence grief trajectories. Intervention strategies encompass cognitive behavioral therapies addressing maladaptive appraisals and avoidance, group based peer support fostering mutual recognition, and digital modalities offering accessible, anonymous engagement. Community and policy-level initiatives emphasize coordinated postvention frameworks, stigma reduction, and integration of services across healthcare, workplace, and educational settings. Identified research gaps include cultural and geographic representation, longitudinal symptom mapping, and evaluation of system-level interventions. Recommendations advocate for multilevel support systems incorporating early screening, flexible therapeutic delivery, family and peer involvement, community engagement, and culturally sensitive adaptations to improve outcomes and reduce chronic impairment among suicide-loss survivors.

**Conclusions:**

Suicide bereavement produces symptoms that overlap with but remain distinct from related disorders, complicating diagnosis and underscoring the need for cultural and contextual sensitivity. Adapted cognitive-behavioral approaches show promise, particularly when addressing stigma and counterfactual thinking, yet research gaps remain in cultural adaptability, long-term efficacy, and inclusion of diverse survivor groups. A comprehensive support system integrating early detection, evidence-based therapies, family and peer networks, digital platforms, and cultural adaptations is needed.

**Disclosure of Interest:**

None Declared

